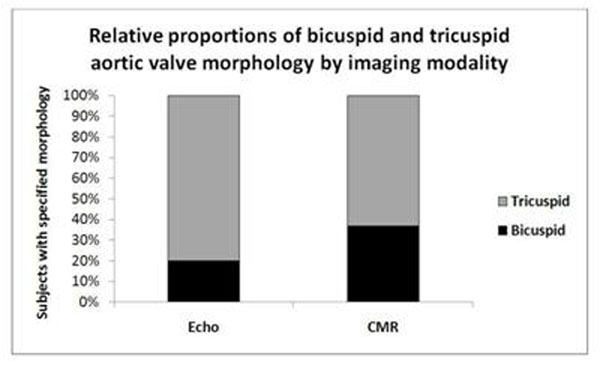# CMR improves identification of aortic valve morphology in aortic stenosis

**DOI:** 10.1186/1532-429X-13-S1-P332

**Published:** 2011-02-02

**Authors:** Sacha Bull, Alex Pitcher, Jane M Francis, Joseph Suttie, Jubin Joseph, Joanna D'Arcy, Bernard Prendergast, Harald Becher, Theo D Karamitsos, Stefan Neubauer, Saul G Myerson

**Affiliations:** 1 Oxford University, Oxford, UK

## Background

The morphology of the aortic valve is of increasing importance in the evaluation of patients with calcific aortic stenosis (AS). The finding of a bicuspid valve can influence the suitability for transcutaneous aortic valve implantation, the attention paid to the proximal aorta, and consideration of family screening. More recent work has also suggested that the type of bicuspid valve can provide important information for planning surgery.

## Methods

65 patients with moderate-severe calcific AS underwent routine clinical trans-thoracic echocardiography (Echo) by an experienced echocardiographer, followed by CMR at 1.5T. Echo was performed using the parasternal short axis view through the aortic valve. CMR was performed in a short axis view through the valve, using SSFP sequences (linear and radial k-space acquisition) and analysis undertaken independently by 2 experienced CMR practitioners. For both imaging modalities, aortic valve morphology was examined over multiple heartbeats, in systole and diastole, and classified according to the Sievers criteria.

## Results

Echo identified 13/65 patients with bicuspid valves. Valve morphology was clearly visualised by CMR in all subjects, and agreed with all 13 identified as bicuspid by Echo. However, CMR also identified an additional 11 subjects with bicuspid aortic valves not identified by Echo (a total of 24/65; p<0.001 vs Echo). The remaining 41 subjects all had clearly tricuspid valves on CMR, and none of these patients had been incorrectly assigned by echo. Overall agreement between CMR and Echo was therefore only moderate (Cohen’s Kappa =0.59).

Valves could be confidently assigned to the correct Sievers’ classification of aortic valve morphology by CMR in all 24 cases. 22/24 subjects had type 1 (asymmetric leaflet size with 1 fused raphe). One subject each had type 0 (symmetric leaflets with no raphes) and type 2 (2 raphes, or unicuspid valve). Of the type 1 valves, 19/22 had right/left cusp fusion, while the remaining 3 had right/non-coronary cusp fusion patterns.

## Conclusions

CMR is a more sensitive method for assessing valve morphology and determining Sievers classification in aortic stenosis than trans-thoracic echocardiography. Echo failed to identify 46% of bicuspid valves (17% of all valves) when compared to CMR. This may be due to the limited excursion of valve leaflets in moderate-severe AS, and the presence of highly echogenic calcifications. The correct identification of aortic valve morphology has implications for choice of valve replacement therapy, management of dilated aortas and family screening.

**Figure 1:**